# Socioeconomic Differences and Trends in the Place of Death among Elderly People in China

**DOI:** 10.3390/ijerph14101210

**Published:** 2017-10-11

**Authors:** Jiaoli Cai, Hongzhong Zhao, Peter C. Coyte

**Affiliations:** 1 School of Economics, Wuhan University of Technology, 122 Luoshi Road, Wuhan 430070, Hubei, China; jiaoli.cai@mail.utoronto.ca (J.C.); zhaohz22@163.com (H.Z.); 2 Institute of Health Policy, Management and Evaluation, University of Toronto, Health Sciences Building, 155 College Street, Suite 425, Toronto, ON M5T 3M6, Canada; 3 Canadian Centre for Health Economics, 155 College Street, Toronto, ON M5T 3M6, Canada

**Keywords:** Chinese elderly, socioeconomic status, place of death, home

## Abstract

China is facing a dramatic aging of its population. Little is known about the factors that influence the place of death and the trends in the place of death for elderly people in China. The purposes of this study were: (1) to examine the impact of the socioeconomic status (SES) on place of death for elderly Chinese residents; and (2) to assess temporal trends in the place of death over the last 15 years. Data were derived from the Chinese Longitudinal Healthy Longevity Survey (CLHLS) (1998–2012). Place-of-death as an outcome was dichotomized into either death at home or death outside the home. Logistic regression analyses were used to examine the impact of SES on place of death. The results showed that, of the 23,098 deaths during the study period, 87.78% occurred at home. The overall trend in home death has increased since 2005. SES was shown to be an important factor affecting place of death. The elderly with higher SES were more likely to die where health resources were concentrated, i.e., in a hospital or other type of institution. Our finding suggests that the trend towards a greater emphasis on death at home may call for the development of more supportive home care programs in China. Our finding also suggests that the socioeconomic differences in the place of death may be related to the availability of or access to health care services.

## 1. Introduction

There has been a recent emphasis in the research literature on studies that assess the determinants of place of death [1,2,3,4,5,6,7]. These studies included population-based studies [1,2], studies that focus on patients with cancer [3,4] and studies that focus on the elderly [5,6,7]. An understanding of the factors that influence the place of death could better inform discussions among healthcare providers, patients and their families regarding patient preferences and the feasibility of dying in the preferred place [8]. This knowledge could also inform policy decisions aimed at improving patients’ likelihood of dying in their preferred place of death [8]. Knowing the trends in the place of death may have implications for the organization of end-of-life care. For instance, health care facilities may be improved and enhanced to support the increased home deaths if the trend in home death is increasing.

Socioeconomic status (SES) has often been found not only to be associated with health and mortality [9,10,11,12,13], but also to be related to place of death [3,6]. Many studies conducted in high-income countries have indicated that people with higher SES are more likely to die at home [6,14,15,16]. This result may be because those with higher SES are better able to access and/or afford preferred home-based services. However, little is known about whether this situation is also applicable to middle-income countries, such as China, where different cultural norms guide health care decision making at the end of life and where community- and home-based end-of-life care services are often sparse. Knowing the relationship between SES and place of death is important for policy decision making that may reduce the socioeconomic differences in the place of death. In high-income countries the socioeconomic differences in the place of death may be related to the availability of resources [6,14,15,16]. Thus we would like to examine whether socioeconomic differences in the place of death also exist in China; and to explore if the differences exist, whether the situation is the same as that in Western countries.

China, a middle-income country, is facing a dramatic aging of its population which has resulted in an increased emphasis on caregiving for the elderly. Death at home has special cultural meanings for the Chinese elderly and their family members. The culture of “fallen leaves return to the roots” in China makes the elderly prefer to die at home. Home brings physical and emotional comfort, a feeling of safety, and a sense of belonging [5,17]. In addition, filial piety has long been advocated in China so that caring for old frail parents is a responsibility of the children [5]. 

Most empirical studies on place of death have been conducted in Western countries [18,19]. Many of the studies that have explored the determinants of place of death have been limited to cross-sectional data [2,6,20]. Although some longitudinal studies have been conducted [4,5,18,21,22], they have generally addressed the place of death for cancer patients [4,18,21,22] or have followed subjects for a relatively short time frame [5]. Consequently, we decided that an interesting contribution to the literature would be to explore the determinants of place of death for the elderly in a non-Western setting. Moreover, given access to a longitudinal survey that followed Chinese elderly over a 15-year period, we were also in a position to examine temporal changes in the place of death for elderly Chinese residents. Thus, the purposes of this study were: (1) to examine the impact of SES on the place of death of elderly Chinese residents; and (2) to assess temporal changes in place of death over the study period.

## 2. Methods

### 2.1. Data

Data were derived from the Chinese Longitudinal Healthy Longevity Survey (CLHLS) (1998–2012). This interview-based survey, which was initiated in 1998, was conducted by the Center for Healthy Aging and Family Studies at Peking University. The survey randomly selected oldest–old Chinese adults (aged 80 years and older) and the younger elderly (aged 65–79) from a randomly selected half of the counties and cities in 22 of China’s 31 provinces. The population in the survey areas constitutes about 85 percent of the total population of China [23]. The CLHLS did not follow the procedure of proportional sampling design, but instead interviewed all centenarians who volunteered to participate in the survey [24]. For each centenarian with a predesignated random code, one nearby octogenarian (aged 80–89), and one nearby nonagenarian (aged 90–99) of a predesignated age and sex were interviewed on a random basis [5]. From 2002, three nearby elders aged 65–79 of predefined age and sex were interviewed in conjunction with every two centenarians. “Nearby” is loosely defined—it could refer to the same village or the same street, if available, or in the same town or in the same sampled county or city [24]. Since 1998, a follow-up face-to-face interview survey was conducted every two or three years. Thus, there were in total six waves of this survey from 1998 till 2012. Specifically, the survey took place in 1998, 2000, 2002, 2005, 2008 and 2011/2012. Those interviewees who were still surviving in the follow-up waves were re-interviewed. Those elderly who were interviewed but subsequently died before the next wave and those who were lost to follow-up were replaced by new interviewees of the same sex and age (or within the same five-year age group). The CLHLS had a response rate higher than 85 percent and relatively low sample attrition [24]. The first two waves focused on the elderly aged 80 years and over. The younger elderly (aged 65–79) were also added from the 2002 wave. The CLHLS contained detailed information on the elderly, such as age, sex, ethnicity, marital status, SES, living arrangement, health conditions and place of death etc. [24,25,26,27]. Data on health status and chronic diseases etc. before dying were collected in interviews with a close family member of the deceased. Data on ethnicity, education and occupation etc. were collected in a previous wave [24]. Details of the CLHLS can be found at the website: http://www.icpsr.umich.edu/icpsrweb/NACDA/studies/36179. Readers can also refer to the book [28] and one paper [25] which describe the survey. 

There were 8589 respondents aged 80 and older in the 1998 baseline survey. There were 3545, 2500 and 2544 elderly people surveyed in Eastern, Middle and Western regions respectively. A total of 4894 younger elderly aged 65–79 were added in the 2002 wave. New interviewees were added from the 2000 wave to replace those who had died or who had been lost to follow-up. Our analysis covered all the waves of the CLHLS, yielding a total of 23,254 deceased respondents aged 65+ during the nearly 15-year study period. Among these deceased respondents, 156 deceased respondents had missing data on their place of death. Thus, our study focused on the remaining 23,098 deceased respondents who had complete information on place of death, representing 99.33% of the original sample. Of these, 3330 died between the 1998 and 2000 waves (including 3001 deaths at home and 329 deaths outside the home), 3259 died between the 2000 and 2002 waves (including 2792 deaths at home and 467 deaths outside the home), 5776 died between the 2002 and 2005 waves (including 5004 deaths at home and 772 deaths outside the home), 5200 died between the 2005 and 2008 waves (including 4528 deaths at home and 672 deaths outside the home), and finally, 5533 died between the 2008 and 2011/2012 waves (including 4950 deaths at home and 583 deaths outside the home).

### 2.2. Measures

#### 2.2.1. Outcome Measure: Place of Death

Place of death was the outcome of interest in this study and was viewed as the dependent variable. This variable was defined as death at home or death outside the home, i.e., the dependent variable was a binary variable with death at home assigned a value of one and death outside the home a value of zero.

#### 2.2.2. Explanatory Variables

The potential predictors of place of death were guided by the Andersen and Newman Behavioural Model of Health Service Utilization [29] and a previous systematic review on factors influencing place of death [30]. We followed Guerriere et al. [18] and used the Andersen and Newman Behavioural Model to guide our work. Place of death represents the site at the end of life where health services are used [31,32]. The Andersen and Newman Behavioural Model considers health service utilization to be a function of three determinants: predisposing factors (such as socio-demographic characteristics), enabling factors (such as income), and needs-based factors (such as functional status) [29]. Gomes and Higginson conducted a systematic review of factors influencing place of death. They generated a conceptual model, which included three sets of factors for place of death: factors related to illness; individual factors; and finally, environmental factors (healthcare input and social support) [30]. Most of the potential predictors involved in these two models were coincident. Thus, in our study, we synthesize these models to yield a single conceptual framework that guided our empirical analysis.

The conceptual framework and the available data resulted in the selection of the following study variables. First, the main explanatory variable in our study was SES. SES was measured by per capita household income, the educational level of the elderly study subject and their spouse, and the occupation of the elderly and their spouse. Per capita household income was divided into quartiles. Educational level was classified into three categories: primary education (including primary school or uneducated), secondary education (including middle or high school), and college or higher education. Occupational status before the age of 60 years was divided into four categories, i.e., professional, technical or managerial personnel; commercial or industrial workers; housekeepers; and farmers. Second, the covariates in our study included age group, sex, ethnic group, residence at death, marital status, living arrangement, functional status, cause of death, whether bedridden or not, types of caregiver, whether receiving timely medical treatment or not, source of medical payment, source of main financial support and the availability of health care services. Functional status was measured by performance in activities of daily living (ADL) (bathing, dressing, indoor transferring, using the toilet, eating and continence) before dying [33] and self-rated health (SRH) status. If the elderly needed help to do any of the six items (bathing, dressing, indoor transferring, using the toilet, eating and continence), they would be regarded as being limited in their ADL, otherwise they would be regarded as not being limited. SRH was divided into three classes: good, fair and poor. Whether the elderly perceived that they received timely medical treatment prior to death was divided into three categories: yes, no or not sick. Causes of death include malignant tumor, cardiovascular disease (CVD) and heart disease, respiratory and digestive disease, accident and natural death. The availability of health care services includes country-specific number of general hospitals, hospital beds per thousand population and ratio of daily occupied beds. Some variables had missing data, but the proportion of missing values was less than 5% for all variables. We followed the work by Guerriere et al. [18] and Yang et al. [34] in dealing with the missing data. Thus for each categorical variable, the observations with missing information were regarded as a separate category. Only the cause of death covariate had a higher proportion of missing values because it was not recorded until the 2005 wave. However, this was not a significant concern, which we have explained in the discussion section. Regional dummy variables were also included. According to China’s economic development and administrative divisions, the 22 provinces were divided into three regional categories, namely Eastern China (The Eastern region included 10 provinces: Beijing, Tianjin, Hebei, Liaoning, Shanghai, Jiangsu, Zhejiang, Fujian, Shandong and Guangdong), Middle China (The Middle region included eight provinces: Shanxi, Jilin, Heilongjiang, Anhui, Jiangxi, Henan, Hubei, and Hunan) and Western China (The Western region included four provinces: Guangxi, Chongqing, Sichuan and Shananxi). Yearly dummy variables were also included to examine the temporal trends in the place of death.

### 2.3. Statistical Analysis

The distribution of the potential predictors by place of death was analyzed. Pearson’s chi-square tests were used to examine whether the differences between places of death in terms of each potential independent variable was statistically significant [35,36]. Univariate logistic regression was applied to examine the crude relationship between SES and place of death. Multivariate logistic regression analysis was employed to analyze the association between SES and place of death after controlling for various covariates. Model fit was evaluated using the Hosmer-Lemeshow test for goodness of fit [37], and collinearity was assessed using the variance inflation factor [38]. All the statistical analyses were performed using Stata (Stata version 13.0 for Mac, StataCorp LP, College Station, TX, USA). All of the results from the univariate and multivariate models were presented as odds ratios (ORs), and 95% confidence intervals (CIs). An OR above one indicates that the specific predictor was more likely to be associated with a home death. We also conducted multivariate regression analysis on the weighted data (The sampling weight variable in the CLHLS dataset was calculated based on the age-sex-urban/rural residence-specific distribution of the population in order to represent the entire elderly population) to perform robustness analysis, and to examine whether our results may generalize to the whole of China. The results from the weighted analysis were reported in the Appendix in order to save space. 

## 3. Results

Table 1 reports the descriptive statistics for the study variables and the distribution of categorical variables by place of death. Most elderly deaths occurred at 90 years of age or older (76.56%). Most of the elderly were female (60.94%). More than 90% of the elderly and their spouses received primary education or never received any formal education. Almost 50% of the elderly and their spouses were farmers. Only 11.05% of the elderly lived alone. Almost 80% of the elderly were limited in their ADLs. Most of the primary caregivers of the elderly were children or grandchildren (82.31%). About 80% of the elderly were financially dependent on their spouse, children or grandchildren. Only a small proportion of the elderly suffered from a specific range of diseases. Based on Pearson’s chi-square tests, the differences between death at home and death outside the home were significantly associated with all the characteristics except the bedridden variable. Of the 23,098 deaths during the study period, 87.78% occurred at home. The proportion of deaths at home rose with increasing age. Elderly women had a higher proportion of deaths at home than men (62.19% vs. 37.81%). The proportion of deaths at home decreased with an increase in SES. Compared with those in rural areas, the elderly in urban areas had a higher proportion of deaths outside the home (76.73% vs. 23.27%).

Table 2 shows the univariate ORs for the likelihood of a home death. Since our focus was to assess the impact of SES on place of death and to examine temporal trends in place of death, our results focus on only the SES and yearly dummy variables. For completeness, all the results from the univariate analysis are reported in the Appendix A (Table A1). All of the SES variables were significantly associated with the place of death. The elderly with a higher per capita household income were less likely to die at home compared with those with a lower per capita household income. The death for the elderly was less likely to occur at home when the elderly or their spouse had received secondary or tertiary education. The death of the elderly was less likely to occur at home when the occupation of the elderly or their spouse was professional, technical or managerial personnel, commercial/industrial workers or housekeepers. Compared with the OR (value 1) in 2000 (as the reference group), the ORs in subsequent years were lower, but grew over the study period. We also calculated the percentage of deaths occurring at home over the study period. Though the proportion of home death decreased from 90.12% in 2000 to 85.67% in 2002, the proportion of home death remained above 85% in each subsequent wave and has increased from 85.67% in 2002 to 89.46% in 2012 (see data section).

Table 3 presents the results of the multivariate regression analysis examining the potential predictors of a home death. Sex, marital status, whether bedridden or not and living arrangement had no statistically significant association with the place of death. Death at home was more likely as age increased. The elderly who were Han nationality (OR, 0.51; 95% CI, 0.39–0.66) and from urban areas (OR, 0.35; 95% CI, 0.32–0.39) were less likely to die at home. The elderly who had higher per capita household income were less likely to die at home. The elderly who died at home were less likely to be those who received secondary or tertiary education, or those whose spouse received secondary or tertiary education. The death of the elderly was less likely to occur at home, when the occupation of the elderly or their spouse was professional, technical or managerial personnel, commercial/industrial workers or housekeepers. These findings suggest that SES was an important factor in determining place of death of the elderly in China. Lower functional status (including poor SRH and being limited in ADL) was associated with dying at home. Compared with deaths from other causes, deaths from malignant tumor, CVD and heart disease, respiratory and digestive disease and accident were less likely to occur at home. The elderly who were cared for by a spouse, children or grandchildren caregivers were more likely to die at home compared with those cared for by other caregivers. The elderly who were able to get timely medical treatment, whose medical costs were covered by public medical care or medical insurance, or who were economically independent were less likely to die at home. An increase in the number of general hospital and hospital beds per thousand population was negatively associated with a home death. Eastern China or Middle China was associated with a lower likelihood of dying at home, compared to Western China. Over time, the likelihood of a home death increased from 2005 onwards.

We also conducted multivariate regression analysis on the weighted data and the results are reported in the Appendix A (Table A2). The main results using weighted analysis were consistent with our results above. The main difference existed in the results of the regional dummy variables. Specifically, regional dummy variables became insignificant in the weighted analysis.

## 4. Discussion

This study is the first to examine the association between SES and place of death for elderly Chinese residents and to assess the temporal trends in the place of death. We found that 87.78% of all deaths occurred at home over the study period (1998 to 2012), and that the overall trend in home death, after controlling for other covariates, has been on the rise since 2005. Having an understanding of the factors that affect the place of death better informs the discussions among healthcare providers, patients and their families regarding patient preferences and the feasibility of dying in the preferred place and may perhaps help explain the incongruence between preferred and actual place of death [8]. Knowing the trends in the place of death may have implications for the organization of end-of-life care. For instance, if most of the patients die at home, health care facilities may be improved and enhanced to support the increased home deaths. An important policy implication from our findings is for the development and promotion of home-based end-of-life care services focusing on the elderly, in order to conform to the trend in home death and to meet the needs of elderly Chinese residents. Presently, China does not have formal end-of-life home care or hospice care on any significant scale [5], but most elderly die at home, which seems a contradictory phenomenon. Home death may be caused by factors such as SES and patients’ preference. As we found in our study, the elderly with lower SES were more likely to die at home. We did not examine the influence of patients’ preference for place of death on the actual place of death, because the information on preference was not available, which is a limitation. However, no matter what causes the high proportion of home death and the increased trend towards home death, the development of home care programs is of important. It can not only improve the situation that China does not have formal end-of-life home care on any significant scale, but also conforms to China’s cultural belief that “fallen leaves return to the roots”.

We found that SES was an important factor associated with place of death. The elderly who had higher SES were less likely to die at home in China, which was contrary to studies in Western countries [2,3,6,14]. While some Western studies identified an inconclusive association between SES and place of death [21,39], the overwhelming consensus in the literature reported that dying at home was more likely among those with higher SES [2,3,6,14]. A population-based cohort study conducted in Ontario, Canada found that higher SES was associated with a greater probability of dying at home [2]. A cross-sectional study conducted in Belgium reported that home death was less frequent in people with lower education [14]. A longitudinal study conducted in England and Wales found that the chance of a home death increased with SES [3]. Another study conducted in Brussels, Belgium reported that residents living in a district with higher SES were more likely to die at home [6]. These results may be due to the fact that in Western countries, those with higher SES are able to get access to home-based care, so they have a greater chance of dying at home. However, the reality of China is different from that of the Western countries. As we mentioned above, China does not have formal end-of-life home-based care services or hospice care services on a large scale [5]. In China, people can get more accessible quality care in hospitals than at home as resources tend to be institutionally concentrated in China [5]. This may be because the elderly with higher SES can afford and also prefer, more quality care in hospitals, such that they receive such care and die in that setting. Those with lower SES were more likely to die at home, which may be because they can not afford the costs associated with hospital care. Therefore, SES may affect place of death through access to health care resources.

We also found some other predictors of a home death. The chance of a home death increased with age, which was consistent with other studies [4,19,20,40], but contrasted with those studies that reported that the younger elderly [3,41], or younger persons [1,22] were more likely to die at home. Gu et al. using data from the CLHLS (1998–2000), found that age was not associated with the place of death [5]. Sex was not a determinant of home death in our study, which is in line with the finding by Gu et al. [5]. However, the effect of sex on place of death was not in line with previous studies. Some studies found that women were more likely to die at home [1,4,19,20,40], while other studies reported that men had a higher chance of dying at home [2]. We found that urban residents were less likely to die at home, which is consistent with previous studies [1,5]. This may be because there existed differences in the structure of the Chinese institutional elderly care system in urban and rural areas [5]. Previous studies in Western countries reported that patients who lived with others were more likely to die at home [3,6,19,42,43], and the social support provided by family members may be a possible explanation for this observation. Despite this, we did not find any evidence to support the association between living arrangement and place of death. We found that lower functional status (poor SRH health and being limited in ADL) was associated with a home death, which is consistent with previous studies [44,45,46,47]. This may be because these elderly wanted to spend the last years of life at home; or they didn’t want to be a financial burden to their family members. Patients with serious disease were less likely to die at home, which is to be expected, since the disease usually warrants hospital admission and is related to high short-term mortality. The elderly who were cared for by spousal caregivers and children were more likely to die at home compared with those cared for by other caregivers. The elderly who received timely medical treatment, who were economically independent and whose medical costs were covered by public medical care or medical insurance had a lower chance of dying at home. This may be because the elderly were able to afford the health care services provided by hospitals or other types of institutions. A higher availability of general hospitals and hospital beds decreased the likelihood of death at home, which is consistent with previous studies [48,49]. Geographic differences in the place of death might be explained by the distribution of health services resources. Compared with the western region, the eastern and middle regions are areas with more hospitals and hospitals beds, which may increase the odds that the elderly people are kept in a hospital for end-of-life care or death. 

There were some limitations associated with our study. First, due to the lack of relevant data, we were unable to control for a range of covariates that may have had an impact on the place of death, e.g., a preference for place of death. Some previous studies have reported that a preference for place of death may be influential in determining the actual place of death [31,50]. The majority of Chinese elderly patients prefer to die at home due to cultural beliefs, so the results might be biased by the patients’ decision, regardless of SES. Thus the findings need to be treated with caution. Despite this absence, we endeavored to incorporate as many potential factors as were allowed for in the data set. Second, it should be noted that this study was focused on where the elderly died instead of what kind of services they received, as we did not have access to data about end-of-life services. Indeed, the place of death was not necessarily the place where the elderly received their end-of-life care. The elderly may have been cared for at home, but may have had a hospital admission (possible due to an acute event) and eventually have died in the hospital. However, a better understanding of where people die has implications for resource allocation for end-of-life care services in hospitals and in the community, thereby improving the quality of end-of-life care. Third, there were many missing values for the cause of death variable. This is because the cause of death was not recorded until the 2005 wave. When we removed this variable from the multivariate model, our results were not changed, suggesting that this was not a significant concern. Fourth, some readers may be concerned about whether the missing data, in general, may affect our results. We have used Little’s chi-squared test [51] and a series of independent *t*-tests [52] to examine whether the missing data impacted our results. This approach separates the missing and the complete cases for a particular variable and uses a *t*-test to examine group mean differences with other variables in the data set. If the cases with observed data were the same as the cases with missing values, then the data that were missing were deemed to be missing at random. We found that the data that were missing were missing at random. Consequently, there was no difference between the missing cases and the complete cases. This suggests that the missing data were not likely to affect our findings. We also conducted our analysis on complete cases, which means that we employed the listwise deletion method, deleting the missing values and performing a regression on observations with non-missing values. Under the condition that the data were missing completely at random, listwise deletion works well, and there is no bias in analyses based on complete cases [53]. We found that the results were consistent with our results above. Fifth, in longitudinal surveys, sample attrition (or data attrition, i.e., respondents lost to a follow-up survey) occurs when previous respondents migrated, refused to participate in the survey, or the address of a previous respondent was not sufficiently detailed. Biased estimates might occur if sample attrition is correlated with some particular attributes, which may result in a lack of generalizability. However, the attrition might not always be a serious problem when associations between variables are the focus of a study, particularly when the proportion of dropouts is not too large [54]. Thus, it is unlikely that there will be significant problems in estimations using CLHLS samples due to the survey’s relatively low sample attrition [24]. For example, the proportion of attrition was 9.6 percent between the 1998 and 2000 waves [24]. In addition, we found by using the *t*-test procedure that the attrition was completely random with respect to the potential factors, so our results were not affected by sample attrition. Specifically, we conducted *t*-tests to examine whether the attrition was random. This approach separates the attrition group and the complete group for a particular variable and uses a *t*-test to examine group mean differences with other variables in the data set. A nonsignificant *t*-test indicates that the attrition was completely random. Last, because the proportion of deaths in hospitals and institutions was small, deaths in hospitals and institutions were together regarded as deaths outside the home.

## 5. Conclusions

We report that the overwhelming majority of elderly deaths in China occur at home and that there has been an increasing trend towards home deaths since 2005. The findings may support the promotion of policies designed to further the development of supportive home-based care in China. SES was identified as an important factor impacting place of death for elderly Chinese residents. Those with higher SES were less likely to die at home, and more likely to die outside the home (in a hospital or other type of institution). We provide a possible explanation for the association between SES and place of death. Access to quality health care services may explain the socioeconomic differences in place of death, i.e., SES may impact the place of death through the availability of and/or access to health services. If this were the case, then the development of home care may attract more people with higher SES to choose to die at home. We could not examine this in our study because the relevant data were not available. However, it is worth verifying in the future. Furthermore, policies should be designed to eliminate socioeconomic differences in place of death, to ensure that the place of death unaffected by socioeconomic status. 

## Figures and Tables

**Table 1:** Descriptive statistics of the total study population.

Variables	% of All Death (*n* = 23,098)	Place of Death (%)		*p*-Value
		Home (*n* = 20,275)	Outside Home (*n* = 2823)	
***Age ^a^***				0.00
65–79	4.73	4.18	8.68	
80–89	18.71	17.53	27.17	
90–99	36.31	36.61	34.11	
100+	40.25	41.68	30.04	
***Sex ^b^***				0.00
Female	60.94	62.19	51.97	
Male	39.06	37.81	48.03	
***Ethnicity ^b^***				0.00
Han	93.18	92.63	97.17	
Minority	6.82	7.37	2.83	
***Marital status ^a^***				0.00
Married	13.18	12.55	17.68	
Not married	86.82	87.45	82.32	
***Per capita household income ^c^***				0.00
Quartile 1	29.73	30.99	20.69	
Quartile 2	21.15	21.84	16.15	
Quartile 3	24.63	24.77	23.66	
Quartile 4	24.49	22.40	39.50	
***Education ^b^***				0.00
Primary/uneducated	94.24	95.44	85.62	
Secondary	4.14	3.44	9.17	
Tertiary	0.99	0.55	4.11	
Missing	0.63	0.57	1.10	
***Spousal Education ^b^***				0.00
Primary/uneducated	90.80	92.19	80.84	
Secondary	3.94	3.33	8.29	
Tertiary	0.90	0.63	2.87	
Missing	4.36	3.85	8.01	
***Occupation ^b^***				0.00
Farmer	49.18	51.89	29.72	
Professional/technical/managerial personnel	4.72	3.50	13.46	
Commercial/industrial worker	25.61	24.99	30.11	
Housekeeper	11.52	11.05	14.84	
Others	8.71	8.31	11.62	
Missing	0.26	0.26	0.25	
***Spousal Occupation ^b^***				0.00
Farmer	51.31	54.24	30.25	
Professional/technical/managerial personnel	4.09	3.30	9.78	
Commercial/industrial worker	26.48	25.94	30.39	
Housekeeper	9.84	9.15	14.77	
Others	6.92	6.46	10.20	
Missing	1.36	0.90	4.61	
***Residence ^a^***				0.00
Urban	46.23	41.98	76.73	
Rural	53.77	58.02	23.27	
***Living arrangement ^c^***				0.00
Lives alone	11.05	10.99	11.47	
Lives with others	88.45	88.54	87.89	
Missing	0.50	0.47	0.64	
***Health status ^c^***				0.00
Good SRH	37.58	37.37	39.07	
Fair SRH	29.94	29.78	31.10	
Poor SRH	16.24	16.16	16.76	
Not able to answer	16.24	16.69	13.07	
***ADLs impaired ^c^***				0.05
Yes	79.14	79.19	78.75	
No	20.57	20.52	20.97	
Missing	0.29	0.30	0.28	
***Bedridden ^c^***				0.72
Yes	28.03	27.96	28.52	
No	71.68	71.76	71.17	
Missing	0.29	0.29	0.32	
***Cause of death ^c^***				0.00
Malignant tumor	1.74	1.46	3.75	
CVD and heart disease	7.92	6.95	14.77	
Respiratory	4.16	3.81	6.66	
Accident	3.98	4.10	3.12	
Natural death	5.52	5.89	2.90	
others	7.14	7.25	6.38	
Missing	69.54	70.54	62.42	
***Primary caregiver ^c^***				0.00
Spouse	3.79	3.57	5.38	
Children/grandchildren	82.31	86.24	54.06	
Others	10.21	6.77	34.96	
No body/do not need care	3.20	2.91	5.21	
Missing	0.49	0.51	0.39	
***Timely medical treatment ^c^***				0.00
Yes	74.26	72.80	84.77	
No	7.24	7.64	4.32	
Not ill	18.17	19.25	10.45	
Missing	0.33	0.31	0.46	
***Financial source ^c^***				0.00
Retirement wage/self–work	10.76	7.86	31.56	
Spouse/children/grandchildren	81.53	86.11	48.64	
Government subsidies/others	7.57	5.88	19.73	
Missing	0.14	0.15	0.07	
***Who paid medical cost ^c^***				0.00
Free public/collective medical care/insurance	14.22	9.93	45.02	
Family	82.04	86.31	51.36	
Others	2.91	2.93	2.76	
Missing	0.83	0.82	0.85	
***Regions ^b^***				0.00
Eastern	38.76	38.09	43.57	
Middle	32.40	32.32	33.01	
Western	28.84	29.60	23.41	
***Year***				0.00
2000	14.42	14.80	11.65	
2002	14.11	13.77	16.54	
2005	25.01	24.68	27.35	
2008	22.51	22.33	23.80	
2011/2012	23.95	24.41	20.65	

SRH, Self-rated health; ADL, Activities of daily living. ^a^ Measured at death; ^b^ Measured at previous wave; ^c^ Measured before death.

**Table 2:** Univariate odds ratios for likelihood of home death.

Variables		OR	LCL	UCL	*p-*Value
Per capita household income	Quartile 2 vs. quartile 1	0.90	0.79	1.03	0.12
	Quartile 3 vs. quartile 1	0.70	0.62	0.79	0.00
	Quartile 4 vs. quartile 1	0.38	0.34	0.42	0.00
Education	Secondary vs. primary/uneducated	0.34	0.29	0.39	0.00
	Tertiary vs. primary/uneducated	0.12	0.09	0.16	0.00
	Missing vs. primary/uneducated	0.47	0.31	0.70	0.00
Spousal education	Secondary vs. primary/uneducated	0.35	0.30	0.41	0.00
	Tertiary vs. primary/uneducated	0.19	0.15	0.26	0.00
	Missing vs. primary/uneducated	0.42	0.36	0.49	0.00
Occupation	Professional/technical/managerial personnel vs. farmer	0.15	0.13	0.17	0.00
	Commercial/industrial worker vs. farmer	0.48	0.43	0.53	0.00
	Housekeeper vs. farmer	0.43	0.38	0.48	0.00
	Others vs. farmer	0.41	0.36	0.47	0.00
	Missing vs. farmer	0.60	0.27	1.33	0.21
Spousal occupation	Professional/technical/managerial personnel vs. farmer	0.19	0.16	0.22	0.00
	Commercial/industrial worker vs. farmer	0.48	0.43	0.53	0.00
	Housekeeper vs. farmer	0.35	0.30	0.39	0.00
	Others vs. farmer	0.35	0.31	0.41	0.00
	Missing vs. farmer	0.11	0.09	0.14	0.00
Year	2002 vs. 2000	0.66	0.56	0.76	0.00
	2005 vs. 2000	0.71	0.62	0.81	0.00
	2008 vs. 2000	0.74	0.64	0.85	0.00
	2011/2012 vs. 2000	0.93	0.81	1.07	0.32

OR, Odds ratio; LCL, Lower 95% confidence limit; UCL, Upper 95% confidence limit. Only SES-related variables and year dummies were reported. SES-related variables were measured at previous waves. SRH, Self-rated health; ADL, Activities of daily living.

**Table 3:** Multivariate odds ratios modelling the odds of home death.

Variables		OR	LCL	UCL	*p*-Value
Age ^a^	80–89 vs. 65–79	1.57	1.28	1.94	0.00
	90–99 vs. 65–79	2.30	1.87	2.82	0.00
	100+ vs. 65–79	2.63	2.12	3.25	0.00
Sex ^b^	Male vs. female	1.06	0.94	1.19	0.35
Ethnicity ^b^	Han vs. minority	0.51	0.39	0.66	0.00
Marital status ^a^	Married vs. not married	1.00	0.85	1.16	0.95
Per capita household income ^c^	Quartile 2 vs. quartile 1	0.89	0.76	1.03	0.10
	Quartile 3 vs. quartile 1	0.79	0.68	0.92	0.00
	Quartile 4 vs. quartile 1	0.46	0.40	0.54	0.00
Education ^b^	Secondary vs. primary/uneducated	0.58	0.42	0.81	0.02
	Tertiary vs. primary/uneducated	0.55	0.39	0.79	0.00
	Missing vs. primary/uneducated	0.91	0.72	1.15	0.44
Spousal education ^b^	Secondary vs. primary/uneducated	0.84	0.69	1.03	0.08
	Tertiary vs. primary/uneducated	0.67	0.46	0.99	0.04
	Missing vs. primary/uneducated	1.08	0.82	1.41	0.58
Occupation ^b^	Professional/technical/managerial personnel vs. farmer	0.80	0.64	1.00	0.05
	Commercial/industrial worker vs. farmer	0.83	0.71	0.96	0.01
	Housekeeper vs. farmer	0.67	0.57	0.79	0.00
	Others vs. farmer	0.65	0.54	0.79	0.00
	Missing vs. farmer	2.14	0.77	5.95	0.14
Spousal occupation ^b^	Professional/technical/managerial personnel vs. farmer	0.53	0.42	0.67	0.00
	Commercial/industrial worker vs. farmer	0.68	0.59	0.79	0.00
	Housekeeper vs. farmer	0.68	0.57	0.80	0.00
	Others vs. farmer	0.66	0.54	0.80	0.00
	Missing vs. farmer	0.42	0.28	0.62	0.00
Residence ^a^	Urban vs. rural	0.35	0.32	0.39	0.00
Living arrangement ^c^	Live alone vs. live with others	1.00	0.88	1.14	0.98
	Missing vs. live with others	0.79	0.42	1.49	0.46
Health status ^c^	Good SRH vs. poor SRH	0.89	0.77	1.02	0.09
	Fair SRH vs. poor SRH	0.92	0.79	1.06	0.23
	Not able to answer vs. poor SRH	0.97	0.82	1.16	0.76
ADLs impaired ^c^	Yes vs. no	1.11	0.97	1.28	0.03
	Missing vs. no	1.23	0.50	3.02	0.65
Bedridden ^c^	Yes vs. no	0.97	0.86	1.09	0.57
	Missing vs. no	1.28	0.53	3.11	0.58
Cause of death ^c^	Malignant tumor vs. others	0.45	0.34	0.60	0.00
	CVD and heart disease vs. others	0.41	0.35	0.49	0.00
	Respiratory vs. others	0.42	0.34	0.53	0.00
	Accident vs. others	0.78	0.60	1.02	0.08
	Natural death vs. others	1.24	0.90	1.70	0.18
	Missing vs. others	1.26	0.42	3.74	0.68
Primary caregiver ^c^	Spouse vs. others	5.83	4.47	7.60	0.00
	Children/grandchildren vs. others	6.01	5.29	6.82	0.00
	No body/do not need care vs. others	2.71	2.13	3.46	0.00
	Missing vs. others	4.27	2.10	8.67	0.00
Timely medical treatment ^c^	Yes vs. no	0.57	0.46	0.71	0.00
	Not ill vs. no	0.92	0.71	1.20	0.55
	Missing vs. no	0.33	0.15	0.71	0.00
Financial source ^c^	Retirement wage/self-work vs. spouse/children/grandchildren	0.58	0.50	0.68	0.00
	Government subsidies/others vs. spouse/children/grandchildren	0.47	0.40	0.56	0.00
	Missing vs. spouse/children/grandchildren	5.82	1.10	30.69	0.04
Who paid medical cost ^c^	Free public/collective medical care/insurance vs. family	0.35	0.31	0.40	0.00
	Others vs. family	1.60	1.19	2.16	0.00
	Missing vs. family	0.75	0.45	1.26	0.28
Availability of health care services ^b^	General hospitals	0.99	0.99	1.00	0.06
	Hospital beds	0.00	0.00	3.88	0.08
	Ratio of daily occupied bed	0.98	0.96	1.00	0.21
Regions ^b^	Eastern vs. western	0.87	0.77	0.99	0.03
	Middle vs. western	0.74	0.65	0.84	0.00
Year	2002 vs. 2000	0.62	0.51	0.77	0.00
	2005 vs. 2000	0.51	0.42	0.63	0.00
	2008 vs. 2000	0.82	0.71	0.94	0.01
	2011/2012 vs. 2000	1.02	0.88	1.17	0.81

OR, Odds ratio; LCL, Lower 95% confidence limit; UCL, Upper 95% confidence limit; SRH, Self-rated health; ADL, Activities of daily living. ^a^ Measured at death; ^b^ Measured at previous wave; ^c^ Measured before death.

## Appendix A

**Table A1:** Univariate odds ratios for likelihood of home death (all the results).

Variables		OR	LCR	UCL	*p*-Value
Age ^a^	80–89 vs. 65–79	1.34	1.14	1.58	0.00
	90–99 vs. 65–79	2.23	1.91	2.61	0.00
	100+ vs. 65–79	2.88	2.46	3.38	0.00
Sex ^b^	Male vs. female	0.66	0.61	0.71	0.00
Ethnicity ^b^	Han vs. minority	0.37	0.29	0.46	0.00
Marital status ^a^	Married vs. not married	0.67	0.60	0.74	0.00
Per capita household income ^c^	Quartile 2 vs. quartile 1	0.90	0.79	1.03	0.12
	Quartile 3 vs. quartile 1	0.70	0.62	0.79	0.00
	Quartile 4 vs. quartile 1	0.38	0.34	0.42	0.00
Education ^b^	Secondary vs. primary/uneducated	0.34	0.29	0.39	0.00
	Tertiary vs. primary/uneducated	0.12	0.09	0.16	0.00
	Missing vs. primary/uneducated	0.47	0.31	0.70	0.00
Spousal education ^b^	Secondary vs. primary/uneducated	0.35	0.30	0.41	0.00
	Tertiary vs. primary/uneducated	0.19	0.15	0.26	0.00
	Missing vs. primary/uneducated	0.42	0.36	0.49	0.00
Occupation ^b^	Professional/technical/managerial personnel vs. farmer	0.15	0.13	0.17	0.00
	Commercial/industrial worker vs. farmer	0.48	0.43	0.53	0.00
	Housekeeper vs. farmer	0.43	0.38	0.48	0.00
	Others vs. farmer	0.41	0.36	0.47	0.00
	Missing vs. farmer	0.60	0.27	1.33	0.21
Spousal occupation ^b^	Professional/technical/managerial personnel vs. farmer	0.19	0.16	0.22	0.00
	Commercial/industrial worker vs. farmer	0.48	0.43	0.53	0.00
	Housekeeper vs. farmer	0.35	0.30	0.39	0.00
	Others vs. farmer	0.35	0.31	0.41	0.00
	Missing vs. farmer	0.11	0.09	0.14	0.00
Residence ^a^	Urban vs. rural	0.22	0.20	0.24	0.00
Living arrangement ^c^	Live alone vs. live with others	0.95	0.84	1.08	0.43
	Missing vs. live with others	0.74	0.44	1.22	0.24
Health status ^c^	Good SRH vs. poor SRH	0.79	0.69	0.90	0.00
	Fair SRH vs. poor SRH	0.86	0.75	0.99	0.04
	Not able to answer vs. poor SRH	0.87	0.73	1.03	0.10
ADLs impaired ^c^	Yes vs. no	1.03	0.93	1.13	0.58
	Missing vs. no	1.07	0.51	2.24	0.86
Bedridden ^c^	Yes vs. no	0.97	0.89	1.06	0.53
	Missing vs. no	0.89	0.44	1.80	0.75
Cause of death ^c^	Malignant tumor vs. others	0.31	0.24	0.39	0.00
	CVD and heart disease vs. others	0.37	0.32	0.42	0.00
	Respiratory vs. others	0.45	0.37	0.53	0.00
	Accident vs. others	1.03	0.82	1.30	0.80
	Natural death vs. others	1.59	1.25	2.01	0.00
	Missing vs. others	0.82	0.75	0.91	0.00
Primary caregiver ^c^	Spouse vs. others	3.42	2.82	4.15	0.00
	Children/grandchildren vs. others	8.24	7.48	9.08	0.00
	No body/do not need care vs. others	2.89	2.37	3.53	0.00
	Missing vs. others	6.74	3.60	12.61	0.00
Timely medical treatment ^c^	Yes vs. no	0.49	0.40	0.59	0.00
	Not ill vs. no	1.04	0.84	1.30	0.71
	Missing vs. no	0.38	0.20	0.71	0.00
Financial source ^c^	Retirement wage/self-work vs. spouse/children/grandchildren	0.84	0.73	0.95	0.01
	Government subsidies/others vs. spouse/children/grandchildren	5.94	5.30	6.66	0.00
	Missing vs. spouse/children/grandchildren	7.01	1.67	29.43	0.01
Who paid medical cost ^c^	Free public/collective medical care/insurance vs. family	0.13	0.12	0.14	0.00
	Others vs. family	0.63	0.50	0.81	0.00
	Missing vs. family	0.58	0.37	0.89	0.01
Availability of health care services ^b^	General hospitals	0.99	0.99	1.00	0.05
	Hospital beds	0.60	0.42	0.85	0.01
	Ratio of daily occupied bed	1.02	1.01	1.04	0.00
Regions ^b^	Eastern vs. western	0.69	0.63	0.76	0.00
	Middle vs. western	0.77	0.70	0.86	0.00
Year	2002 vs. 2000	0.66	0.56	0.76	0.00
	2005 vs. 2000	0.71	0.62	0.81	0.00
	2008 vs. 2000	0.74	0.64	0.85	0.00
	2011/2012 vs. 2000	0.93	0.81	1.07	0.32

OR, Odds ratio; LCL, Lower 95% confidence limit; UCL, Upper 95% confidence limit; SRH, Self-rated health; ADL, Activities of daily living. ^a^ Measured at death; ^b^ Measured at previous wave; ^c^ Measured before death.

**Table A2:** Multivariate odds ratios modelling the odds of home death (weighted analysis).

Variables		OR	LCR	UCL	*p*-Value
Age ^a^	80–89 vs. 65–79	1.50	1.17	1.92	0.00
	90–99 vs. 65–79	2.08	1.57	2.75	0.00
	100+ vs. 65–79	2.49	1.83	3.39	0.00
Sex ^b^	Male vs. female	1.10	0.87	1.40	0.43
Ethnicity ^b^	Han vs. minority	0.38	0.23	0.66	0.00
Marital status ^a^	Married vs. not married	0.90	0.68	1.17	0.42
Per capita household income ^c^	Quartile 2 vs. quartile 1	0.81	0.70	0.94	0.00
	Quartile 3 vs. quartile 1	0.72	0.61	0.82	0.00
	Quartile 4 vs. quartile 1	0.51	0.37	0.70	0.00
Education ^b^	Secondary vs. primary/uneducated	0.60	0.40	0.85	0.02
	Tertiary vs. primary/uneducated	0.30	0.16	0.57	0.00
	Missing vs. primary/uneducated	0.49	0.17	1.41	0.18
Spousal education ^b^	Secondary vs. primary/uneducated	0.54	0.38	0.77	0.00
	Tertiary vs. primary/uneducated	0.29	0.14	0.60	0.00
	Missing vs. primary/uneducated	1.31	0.71	2.43	0.39
Occupation ^b^	Professional/technical/managerial personnel vs. farmer	0.65	0.52	0.80	0.00
	Commercial/industrial worker vs. farmer	0.75	0.66	0.87	0.00
	Housekeeper vs. farmer	0.58	0.43	0.77	0.00
	Others vs. farmer	0.76	0.49	1.19	0.23
	Missing vs. farmer	2.02	0.47	8.66	0.35
Spousal occupation ^b^	Professional/technical/managerial personnel vs. farmer	0.55	0.33	0.92	0.02
	Commercial/industrial worker vs. farmer	0.70	0.50	0.97	0.03
	Housekeeper vs. farmer	0.74	0.53	1.04	0.07
	Others vs. farmer	0.72	0.47	1.10	0.13
	Missing vs. farmer	0.69	0.28	1.69	0.42
Residence ^a^	Urban vs. rural	0.45	0.36	0.57	0.00
Living arrangement ^c^	Live alone vs. live with others	0.91	0.79	1.04	0.18
	Missing vs. live with others	0.61	0.13	2.89	0.53
Health status ^c^	Good SRH vs. poor SRH	0.82	0.62	1.09	0.10
	Fair SRH vs. poor SRH	0.97	0.73	1.30	0.86
	Not able to answer vs. poor SRH	1.29	0.86	1.94	0.22
ADLs impaired ^c^	Yes vs. no	1.41	1.04	1.91	0.03
	Missing vs. no	1.97	0.30	12.82	0.48
Bedridden ^c^	Yes vs. no	0.92	0.68	1.24	0.59
	Missing vs. no	0.28	0.06	1.37	0.12
Cause of death ^c^	Malignant tumor vs. others	0.56	0.32	0.97	0.04
	CVD and heart disease vs. others	0.48	0.32	0.72	0.00
	Respiratory vs. others	0.57	0.35	0.92	0.02
	Accident vs. others	0.37	0.20	0.67	0.00
	Natural death vs. others	1.48	0.74	2.96	0.27
	Missing vs. others	1.24	0.79	1.95	0.36
Primary caregiver ^c^	Spouse vs. others	9.40	5.78	15.28	0.00
	Children/grandchildren vs. others	7.17	5.17	9.95	0.00
	No body/do not need care vs. others	4.68	2.73	8.05	0.00
	Missing vs. others	5.81	1.85	18.21	0.00
Timely medical treatment ^c^	Yes vs. no	0.67	0.43	1.03	0.07
	Not ill vs. no	0.69	0.40	1.19	0.18
	Missing vs. no	1.33	0.13	13.16	0.81
Financial source ^c^	Retirement wage/self-work vs. spouse/children/grandchildren	0.50	0.37	0.69	0.00
	Government subsidies/others vs. spouse/children/grandchildren	0.43	0.29	0.65	0.00
	Missing vs. spouse/children/grandchildren	2.96	0.58	15.05	0.19
Who paid medical cost ^c^	Free public/collective medical care /insurance vs. family	0.35	0.25	0.49	0.00
	Others vs. family	1.60	0.71	3.63	0.26
	Missing vs. family	0.70	0.23	2.10	0.52
Availability of health care services ^b^	General hospitals	0.99	0.99	1.00	0.03
	Hospital beds	0.00	0.00	0.06	0.03
	Ratio of daily occupied bed	1.36	0.90	2.06	0.15
Regions ^b^	Eastern vs. western	1.18	0.89	1.55	0.25
	Middle vs. western	0.93	0.70	1.22	0.58
Year	2002 vs. 2000	0.48	0.33	0.69	0.00
	2005 vs. 2000	0.41	0.27	0.61	0.00
	2008 vs. 2000	0.67	0.52	0.87	0.00
	2011/2012 vs. 2000	0.82	0.61	1.10	0.18

OR, Odds ratio; LCL, Lower 95% confidence limit; UCL, Upper 95% confidence limit; SRH, Self-rated health; ADL, Activities of daily living. ^a^ Measured at death; ^b^ Measured at previous wave; ^c^ Measured before death.
